# Mouse *Protocadherin-1* Gene Expression Is Regulated by Cigarette Smoke Exposure *In Vivo*

**DOI:** 10.1371/journal.pone.0098197

**Published:** 2014-07-03

**Authors:** Henk Koning, Antoon J. M. van Oosterhout, Uilke Brouwer, Lisette E. den Boef, Renée Gras, Marjan Reinders-Luinge, Corry-Anke Brandsma, Marco van der Toorn, Machteld N. Hylkema, Brigitte W. M. Willemse, Ian Sayers, Gerard H. Koppelman, Martijn C. Nawijn

**Affiliations:** 1 Pediatric Pulmonology and Pediatric Allergology, Beatrix Children’s Hospital, University of Groningen, University Medical Center Groningen (UMCG), Groningen, the Netherlands; 2 Laboratory of Allergology and Pulmonary Diseases, Department of Pathology and Medical Biology, University of Groningen, University Medical Center Groningen (UMCG), Groningen, the Netherlands; 3 Department of Pathology and Medical Biology, University of Groningen, University Medical Center Groningen (UMCG), Groningen, the Netherlands; 4 GRIAC research institute, University of Groningen, University Medical Center Groningen (UMCG), Groningen, the Netherlands; 5 Division of Therapeutics and Molecular Medicine, Nottingham Respiratory Biomedical Research Unit, University of Nottingham, Queen’s Medical Centre, Nottingham, United Kingdom; University of Rochester Medical Center, United States of America

## Abstract

Protocadherin-1 (*PCDH1*) is a novel susceptibility gene for airway hyperresponsiveness, first identified in families exposed to cigarette smoke and is expressed in bronchial epithelial cells. Here, we asked how mouse Pcdh1 expression is regulated in lung structural cells *in vivo* under physiological conditions, and in both short-term cigarette smoke exposure models characterized by airway inflammation and hyperresponsiveness and chronic cigarette smoke exposure models. *Pcdh1* gene-structure was investigated by Rapid Amplification of cDNA Ends. Pcdh1 mRNA and protein expression was investigated by qRT-PCR, western blotting using isoform-specific antibodies. We observed 87% conservation of the *Pcdh1* nucleotide sequence, and 96% conservation of the Pcdh1 protein sequence between men and mice. We identified a novel *Pcdh1* isoform encoding only the intracellular signalling motifs. Cigarette smoke exposure for 4 consecutive days markedly reduced *Pcdh1* mRNA expression in lung tissue (3 to 4-fold), while neutrophilia and airway hyperresponsiveness was induced. Moreover, *Pcdh1* mRNA expression in lung tissue was reduced already 6 hours after an acute cigarette-smoke exposure in mice. Chronic exposure to cigarette smoke induced loss of Pcdh1 protein in lung tissue after 2 months, while Pcdh1 protein levels were no longer reduced after 9 months of cigarette smoke exposure. We conclude that *Pcdh1* is highly homologous to human *PCDH1*, encodes two transmembrane proteins and one intracellular protein, and is regulated by cigarette smoke exposure *in vivo*.

## Introduction

Asthma is a complex disease caused by gene-gene and gene-environment interactions [1]. Many asthma susceptibility genes have been identified, several of which are expressed in the airway epithelium [2]. The airway epithelium in asthma has a disrupted barrier function [3] and an impaired repair response upon injury [4], [5] that might contribute to airway remodelling. In addition, the airway epithelium of asthmatics shows an enhanced immune response towards harmful agents (allergens or viruses) by secreting increased amounts of pro-inflammatory mediators such as IL-33, CCL20, GM-CSF or TSLP [6]–[9]. Polymorphisms in asthma susceptibility genes expressed by airway epithelial cells, affecting the level or regulation of their expression [10], might therefore contribute to both altered barrier function of airway epithelial cells and to enhanced induction of an immune response upon airway epithelial injury [11].

Previously, we identified protocadherin-1 (*PCDH1*) as a novel susceptibility gene for airway hyperresponsiveness (AHR) in asthma families [12]. Interestingly, a strong linkage signal of *PCDH1* with asthma and AHR was observed in families exposed to environmental tobacco smoke (ETS). *PCDH1* encodes for two main isoforms: a 3 exon and a 5 exon isoform that are expressed in the airway epithelium [12]. In addition a putative third isoform was identified that lacks exon 1 and part of exon 2 [13]. Both main isoforms encode a protein containing an extracellular domain with seven cadherin repeats, a transmembrane domain, and an intracellular domain containing several Serine and Tyrosine residues, that have been found to be subject to phosphorylation [14], [15]. The third isoform only contains two extracellular cadherin repeats and the shared intracellular domain. In addition, both isoforms 2 and 3 encode an additional intracellular domain containing three intracellular conserved motifs (CM1–CM3), of which CM3 is the binding motif for protein phosphatase 1 alpha (PP1α) [16], [17]. We previously reported complex splicing patterns of *PCDH1* regarding the expression of intracellular conserved motifs, and observed a marked upregulation of PCDH1 during mucociliary differentiation of primary bronchial epithelial cells [13].

In mouse, *Pcdh1* mRNA expression was identified in several adult tissues (brain, kidney, heart, lung and uterus), but highest expression was observed in lung [18]. During mouse embryogenesis, *Pcdh1* mRNA expression in lung was restricted to mesenchyme and blood vessels, and was not detected in the bronchial epithelium. Similar to the human situation, two main transcripts were identified in the mouse, as well as a variant displaying variation in expression of conserved motifs [16].

As *PCDH1* was originally identified as a susceptibility gene for AHR in families exposed to cigarette smoke and encodes an adhesion molecule that is expressed in the airway epithelium, we hypothesize that environmental exposures such as cigarette smoke may affect PCDH1 levels or function in the airways. Currently, detailed knowledge about Pcdh1 expression in lung structural cells and its regulation by environmental exposures *in vivo* is unknown. Therefore, we aimed to investigate the expression and regulation of Pcdh1 under basal conditions *in vivo* and in both short-term and chronic cigarette smoke exposure mouse models.

## Materials and Methods

### Animal Models

BALB/c and A/J mice (6 to 8 weeks, n = 61 for BALB/c and n = 28 for A/J in total) were purchased from Charles River Laboratories (L’Arbresle-Cedex, France), housed in individually ventilated cages, kept under specific pathogen-free conditions and maintained on a 12 h light-dark cycle, with food and water ad libitum. Experiments were approved by the Institutional Animal Care and Use Committee of the University of Groningen (The Netherlands), and carried out following (inter-)national welfare regulations.

*Sub-chronic and acute cigarette smoke (CS) exposure models.*

BALB/c mice were exposed to gaseous-phase CS from Kentucky 3R4F research reference cigarettes (Tobacco Research Institute, University of Kentucky, Lexington, USA). Each cigarette was smoked without filter in 5 minutes at a rate of 5 L/hr in a ratio with 60 L/hr air using whole body exposure. Gaseous-phase CS was directly distributed inside 6-liter perspex boxes. In this study we employed two whole-body CS-exposure models:

*Sub-chronic CS exposure model:* Female mice (n = 8 per group) were exposed to CS of 1–5 cigarettes for 5 days or filtered air each morning and afternoon, using a peristaltic pump as described previously [19]. Mice were sacrificed 2 h after the last CS exposure. The results were replicated in a second independent experiment (n = 8 per group) that was performed in the same way, with the exception that no Bronchial Alveolar Lavage (BAL)-fluid was isolated. In total 32 mice were used for these two independent experiments.

*Acute CS exposure model:* Male mice were exposed to CS of 10 cigarettes (n = 8) in 1.5 h or filtered air (n = 5), and were sacrificed 6 h after the last CS exposure. This experiment was performed once, with 13 mice in total.

From all mice, BAL-fluid was isolated and the smallest lung lobe was stored at −80°C for RNA or protein isolation. BAL was obtained, by lavaging through a tracheal cannula with five 1 ml aliquots of saline of 37°C. Differential BAL cell counts (3 times 100 cells) were obtained from cytospin preparations stained with Diff-Quick (Merz & Dade A.G., Dudingen, Switzerland).

Airway responsiveness (AHR) was assessed in a separate group of sub-chronic CS exposed mice at day 5, by omitting the final smoke exposure (n = 8 per group, 16 mice in total). AHR was determined by measuring airway resistance in response to i.v. administration of increasing doses of methacholine (acetyl-b-methylcholine chloride, Sigma-Aldrich, St. Louis, MO), using a computer-controlled small-animal ventilator (Flexivent; SCIREQ, Montreal, Quebec, Canada) as described previously [20].

*Chronic CS exposure model:*

Female A/J mice were exposed to cigarette smoke from 2R1 reference cigarettes twice daily (2 cigarettes/session, 10 puffs/cigarette), 5 days per week by nose-only exposure as described previously [32]. After 2 or 9 months of cigarette smoke exposure, mice were sacrificed. The right lung lobe was snap frozen for further analyses.

### RNA purification, Rapid amplification of cDNA ends (RACE) and quantitative (q)RT-PCR

RNA was isolated from 50–100 mg of mouse lung tissue homogenized in 1 ml of TriReagent (MRC, Cincinnati, OH). gDNA traces were removed enzymatically, followed by purification using RNeasy Mini Kit (QIAGEN). RNA concentration and integrity was determined by Nanodrop measurements (ND-1000 spectrophotometer, Isogen Lifesciences, de Meern, Netherlands).

RACE was performed on RNA purified from lung tissue according to the manufacturer’s instructions (GeneRacer-RLM-RACE Kit, Invitrogen, Carlsbad, USA), and as described previously [13] using the primers described in Table 1. RACE was performed from *Pcdh1* exon 1 for 5′ transcripts and from exon 3 for 3′transcripts. Furthermore, additional 5′RACE experiments from exon 3 were performed in order to identify a homolog of the putative human isoform 3 [13]. PCR products were TOPO-cloned and *Pcdh1* transcripts were sequenced (StarSEQ, Mainz, Germany) as described previously [13]. Pcdh1 mRNA and protein sequences were compared to human PCDH1 using ClustalW (http://www.ebi.ac.uk/Tools/msa/clustalw2/).

**Table 1:** Pcdh1 RACE and qPCR primer sequences.

*Pcdh1* RACE primer	Sequence (5′-3′)	Corresponding GeneRacer primer	Sequence (5′-3′)
5′RACE 301Rev	GAGGTCTCTGTGGTGAAAATGTCT	GeneRacer 5′ primer	CGACTGGAGCACGAGGACACTGA
5′RACE 162Rev	GTTCCTCTGGCACCTTGTATACTACC	GeneRacer 5′ nested primer	GGACACTGACATGGACTGAAGGAGTA
3′RACE FW1	GCCCAGGAGCTGCAGGATCCAT	GeneRacer 3′ primer	GTCGTCAACGATACGCTACGTAACG
3′RACE FW2	GAAACACCCTCTAGCAAGTCATCCT	GeneRacer 3′ nested primer	CGCTACGTAACGGCATGACAGTG
5′RACE Ex3 Rev3	GCTGTGATGGATCCTGCAGCTCCT	GeneRacer 5′ primer	CGACTGGAGCACGAGGACACTGA
5′RACE Ex3 Rev2	ACTGGTGGCCGAGAAGGTGACA	GeneRacer 5′ nested primer	GGACACTGACATGGACTGAAGGAGTA
qPCR assay	Forward (5′–3′)	Probe (FAM-NFQ labelled)	Reverse (5′-3′)
M-1B–Ex1	CTCAGCATGCGCAGAAGAAAA	ACTGTTCTCCTGATTCTGG	CCTCAGAGGCCCCATCCT
Ex2-Ex3	GAGCAGTACTCCGACTACAG	CCAGCAAGCAGTTACCTCAC	TAGAGGGTGTTTCCGATTCC

*Ex = exon; Rev = reverse; FW = Forward.*

Expression levels of *Pcdh1* transcripts were determined by qRT-PCR as described previously [13], using ABI primer-probe sets. In brief, two microgram of RNA was reverse transcribed in 20 µl reaction volume using Omniscript™ reverse transcriptase (QIAGEN Benelux BV, Venlo, the Netherlands). *Pcdh1* exon 1–2 (Mm01264041_m1, Applied Biosystems Europe BV, Nieuwekerk A/D IJssel, the Netherlands), *Pcdh1* exon 3–4 and *Pcdh1* exon m-1B (Custom assays, Applied Biosystems, Table 1 for primer sequences) expression was determined relative to the most stable combination of four housekeeping genes (*HPRT1* (Mm01545399_m1), *Pgk1* (Mm01225301_m1), *B2m* (Mm00437762_m1), *Ipo8* (Mm01255158_m1), Applied Biosystems). Data was analyzed using SDS2.3 software by applying the ΔΔCt-method (Applied Biosystems User Bulletin 2). As four house-keeping genes were used, for normalization the best combination of house-keeping genes was determined by using the Normfinder applet [21]. We calculated relative expression levels as follows: First the cycle threshold value (Ct-value) of the average of the combination of most stable house-keeping genes was subtracted from the Ct-values of our *Pcdh1*-assays (formula 1). Next, we converted the ΔCt-value to expression level (EL; formula 2), and normalized the expression levels relative to the average of the control group (formula 3). In formulas: 1). ΔCt*_Pcdh1_* = Ct*_Pcdh1_*−Ct*_HKG_*_-*average*_; 2). EL*_Pcdh1_* = 2^−ΔCt*Pcdh1*^; 3). Relative EL = EL*_Pcdh1_*/EL*_Pcdh1-control_*.

### Cloning and overexpression of Pcdh1 isoform 3 in BEAS2B bronchial epithelial cells

*Cloning:* The human cDNA sequence corresponding to the mouse isoform 3 sequence obtained by RACE PCR experiments was amplified from the isoform 2 plasmid (pCMV-XL6-iso2, TC109554, Origene, Rockville, USA) with forward primer CCGCGAATTCATTCTCTTTGGTGTGGTGG and reverse primer CACTGGAGTGGCAACTTC by using Phusion™ High Fidelity PCR Kit (F-553S, Finnzymes, Fisher Scientific, Landsmeer, The Netherlands), followed by subcloning of the PCR product into the pCMV-XL6 expression vector (Origene, USA). The resulting pCMV-XL6-Pcdh1-Iso3 plasmids were sequenced (StarSeq Sequencing, Mainz, Germany).

**Figure 1:**
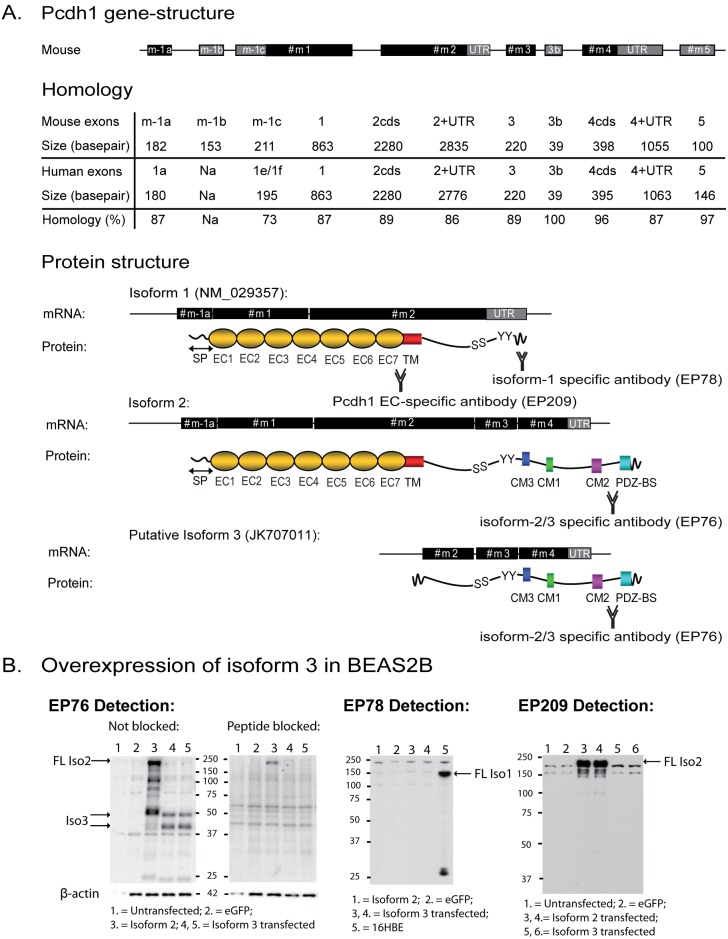
Pcdh1 gene-structure and isoforms. (A) Two novel exons were detected at the 5′end of PCDH1, and one novel exon on the 3′end of Pcdh1. Mouse Pcdh1 exons share high homology with human PCDH1 exons. Corresponding isoforms (isoforms 1, 2 and putative isoform 3) and their protein structures are depicted. Only isoform 2 and putative isoform 3 contain evolutionary conserved motifs (CM1–3). Two antibodies were generated against sequences in the intracellular tail of both isoform 1 (EP78) and isoform 2 (EP76) of Pcdh1. An antibody directed against the extracellular domains of Pcdh1 (EP209) was generated previously [12]. (B) Western blot of lysates of BEAS2B cells overexpressing the open reading frame of Pcdh1 isoform 3, using antibody EP76 for detection as indicated (left hand panel) including a pre-incubation with the immunizing peptide (‘Peptide blocked’) as a control for specificity (right hand panel). Western blots using EP78 and EP209 antibodies for detection are also included as indicated. cds = coding sequence; UTR = Untranslated region; SP = Signal peptide (amino-acid (aa) 1–57); EC = Extracellular Cadherin domain; TM = Transmembrane domain; CM = Conserved Motif; PDZ-BS = PDZ-domain Binding Site; S = Serine residue; Y = Tyrosine residue; EP209 = Binding site for antibody directed against the extracellular domain of Pcdh1; EP78 = Binding site for antibody directed against a specific intracellular sequence of isoform 1, encoded by exon 2; EP76 = Binding site for antibody directed against the intracellular domain present in isoforms 2 and 3 and encoded by exon 4; FL = full length. Genbank accession numbers of novel transcripts are provided in Table 2.

*Overexpression:* BEAS2B cells were grown as described previously [22], seeded into 24-well plates, and transfected at a confluency of 60–80% with 0.5 µg of eGFP (expression control, pEGFP-C1, Westburg BV, Leusden, The Netherlands), full-length Isoform 2 (pCMV-XL6-Pcdh1-iso2), or Isoform 3 plasmid (pCMV-XL6-Pcdh1-iso3), using Fugene HD transfection reagent according to the manufacturer’s procedures (Roche Diagnostics, Almere, the Netherlands). Cells were harvested 48 h after transfection using leammli buffer (2x leammli: 4% SDS; 20% glycerol; 10% β-mercaptoethanol; 0.004% bromphenol blue; 0.125 M Tris HCl; pH 6.8), boiled for 5 min, and analysed for PCDH1 protein expression by western blotting using the antibody specific for the intracellular domain encoded by exon 4 (EP76, Figure 1A) [13].

### Detection of Pcdh1 protein expression levels by western blotting

*Protein isolation:* Frozen lungs were homogenized in 5 µl/mg lysis buffer (1% Triton-X 100, 150 mM NaCl, 5 mM MgCl_2_, 10 mM HEPES, 1∶500 Protease Inhibitor Cocktail (P8340, Sigma-Aldrich, Zwijndrecht, the Netherlands)) using a tissue homogenator (T10 basic, ULTRA-TURRAX, IKA, Staufen, Germany). Subsequently lung homogenates were boiled for 10 minutes with 2x laemmlli buffer and stored at −80°C.

**Figure 2:**
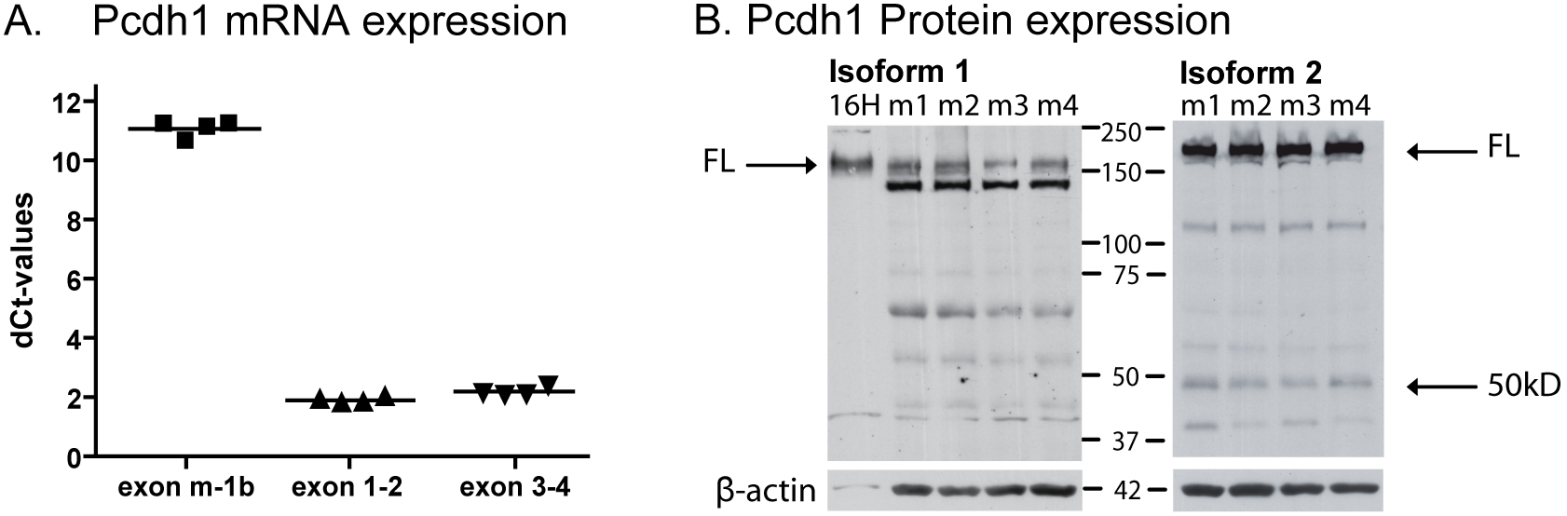
Basal Pcdh1 mRNA and protein expression in lung. (A) mRNA expression levels of exon m-1b, exon 1–2 and exon 3–4 containing transcripts in naive mouse lung of four mice. (B) Western blot of mouse lung homogenate using the antibody directed against the intracellular domain of Pcdh1 isoform 1 (EP78), and the antibody directed against the intracellular domain encoded by exon 4 of Pcdh1 (isoform 2, EP76) (see arrows for the Pcdh1 specific bands), with β-actin as loading control. m1 = lung homogenate of mouse number 1; FL = full length.

*Western blotting:* Lung tissue homogenates were separated on 7.5% acrylamide SDS-page gels (Bio-Rad Laboratories BV, Veenendaal, the Netherlands). Detection of Pcdh1 protein was performed using antibodies that were designed against isoform-specific peptide sequences in the intracellular tail of both human *PCDH1* variants as described previously [13] (Figure 2A). These antibodies showed cross-reactivity with mouse *Pcdh1* due to high homology of the immunizing peptide sequences: (QPFQLSTPQPLPHPYH, EP78, 86% conserved; SPSPPEDRNTKTAPV, EP76, 100% conserved) (See Figure 1A for binding sites of antibodies). Antibodies were affinity column purified against immunizing peptides (Eurogentec, Liege, Belgium), and were validated by blocking experiments using a pre-incubation period of 30 min at 37°C with 10x molar excess of immunizing peptides or H_2_O.

Pcdh1 bands on western blots were visualized using Goat-anti-Rabbit-Peroxidase (GARPO, Daco, Heverlee, Belgium) as secondary antibody, and as tertiary step Rabbit-anti-Goat-Peroxidase (RAGPO, Dako, Heverelee, Belgium) was used. Next, blots were incubated with Luminol-reagent, and subsequently analysed using Fuji Medical X-Ray films (Fuji Photo Film Gmbh, Germany), followed by densitometric analysis using Quantity One Software v4.6.2 (Bio-Rad), relative to the β-actin loading control (sc-47778, Santa-Cruz Biotechnology, Heidelberg, Germany).

### Statistical analysis

Differences in mRNA or protein expression levels were statistically tested by Mann-Whitney tests (GraphPad v4.0, La Jolla, USA). Experimental outliers were determined by using the Grubbs analysis (http://www.graphpad.com/quickcalcs/Grubbs1.cfm). Differences in airway hyperresponsiveness between smoke- and air-exposed mice were determined by testing for differences in the area under the curve (AUC) using GraphPad software.

## Results

### Pcdh1 expression in the mouse lung

To allow an accurate investigation of *Pcdh1* expression regulation in the mouse lung, we first determined the *Pcdh1* gene-structure by RACE experiments. Compared to the published mouse *Pcdh1* gene-structure [16], we detected two novel exons on the 5′ end of *Pcdh1* (exon m-1b and m-1c, Figure 1A), two novel exons on the 3′ end (exon 3b and exon 5), as well as major variation in exon 4, containing the conserved CM2-domain and PDZ-BS motifs (Figure 1A). Exon 3b is a short 39 basepair exon encoding a premature stop codon, which is completely conserved between men and mice (See Table 2 for corresponding Genbank accession numbers). Remarkably, mouse *Pcdh1* exons had high homology to human exons resulting in a 87% homology at the nucleotide level (Figure 1A) and a 96% homology on protein level (Figure S1). Previously we detected an isoform lacking several extracellular domains in human bronchial epithelial cells [13]. We therefore performed 5′RACE experiments from exon 3 in mouse lung. Interestingly, we identified an additional transcript starting from within exon 2, containing a CTG Kozak-sequence (isoform 3, Genbank accession JK707011), and putatively encoding a protein product with an in-frame translation initiation site at amino-acid no 870 of the full-length Pcdh1 isoform 2 protein (Genbank accession NP_115796). Overexpression of the corresponding 5′-truncated open reading frame in BEAS2B cells induced 40- and 50 kD Pcdh1 proteins. Both protein products were specifically detected by the EP76-antibody as intensity of these bands clearly diminished after blocking with immunizing peptide, and were not detected with the EP78 and EP209 antibodies (Figure 1B). These results show that in mouse lung a third, intracellular Pcdh1 isoform is expressed that does not contain the extracellular and transmembrane domains, but retains all intracellular domains also present in Pcdh1 isoform 2 (Figure 1A).

**Table 2:** Genbank accession numbers of novel *Pcdh1* transcripts.

RACE reaction	*Protocadherin-1* Sequence	GenBank Accession number
Mm 5′RACE exon 1	Exon m-1b - 1	JK707005
Mm 5′RACE exon 1	Exon m-1c - 1	JK707006
Mm 5′RACE exon 1	Exon m-1c - 1 (short exon)	JK707007
Mm 3′RACE exon 3	Exon 3-3b-4	JK707008
Mm 3′RACE exon 3	Exon 4 including gap	JK707009
Mm 3′RACE exon 3	Exon 4-5	JK707010
Mm 5′RACE exon 3	Exon 2-3 (Isoform 3)	JK707011
Hs 3′RACE exon 3	Exon 3-3b-4–5	JK707004

*Mm = Mus musculus; Hs = Homo sapiens.*

We next investigated basal expression of *Pcdh1* mRNA and protein levels in mouse lung tissue. We observed very low levels of *Pcdh1* using the exon m-1b/exon-1 specific qRT-PCR assay, while much higher expression levels of *Pcdh1* were detected using exon 1–2 and exon 3–4 specific assays (Figure 2A). On western blot of whole mouse lung homogenate, we observed one specific band of 150 kD and a non-specific band of 140 kD, using an antibody generated against the intracellular tail of the PCDH1 isoform 1 (EP78, Figure 2B). In contrast, we observed a 50 kD and 170 kD bands using a specific antibody generated against the intracellular tail present in PCDH1 isoforms 2 and 3 (EP76, Figure 2B).

### Short term smoke exposure down regulates Pcdh1 expression levels

**Figure 3:**
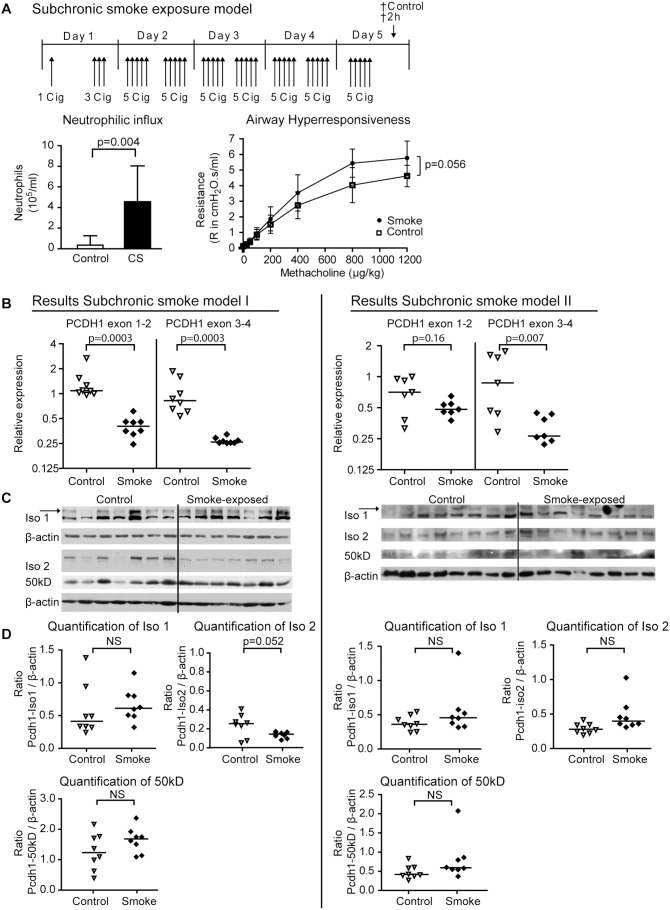
Pcdh1 mRNA expression levels decrease after sub chronic smoke exposure. (A) In the sub chronic smoke exposure model, mice were exposed to 1 and 3 cigarettes or air at day 1, followed by 5 cigarettes at day 2–5 or air. 2 h after the last cigarette both smoke and air exposed mice were sacrificed. Neutrophils in the bronchoalveolar lavage fluid, and airway hyperresponsiveness to methacholine were determined. Error bars show Standard Deviation. Two independent experiments are shown individually. (B) Pcdh1 exon 1–2 and 3–4 transcript levels were determined in lungs of air exposed (open symbols) and smoke exposed (closed symbols) mice. (C) Pcdh1 protein levels were determined by western blot of lung homogenate of air (control) and smoke exposed mice. Pcdh1 isoform 1 (arrow indicates the specific band; EP78-antibody), full-length isoform 2 (EP76-antibody) and 50 kD (EP76-antibody) levels are shown, with β-actin as loading control. (D) Densitometric quantification of Pcdh1 expression levels of full-length isoform 1 and 2, and the 50 kD fragment, relative to β-actin. Cig = cigarette; † = time-point at which mice are sacrificed.

Given the initial linkage of *PCDH1* to AHR and asthma in cigarette smoke exposed families defined by a significant smoking history of the asthma proband of more than 5 packyears [12], we investigated whether *Pcdh1* expression was regulated in a sub-chronic (5 days) smoke exposure model (Figure 3A). This model induced a neutrophillic influx into the airways (Figure 3A), a feature of sub-chronic smoke exposure models [19], [23], as well as a trend towards AHR to methacholine (p = 0.056, Figure 3A).

In CS-exposed mice, we observed significantly lower expression of *Pcdh1* transcripts encoding exon 1–2 (3.9 fold, p = 0.0003) and exon 3–4 (3.3-fold, p = 0.0003) in total lung (Figure 3B, left panel). Next, we investigated whether this was reflected at the protein level in total mouse lung homogenate. No differences in Pcdh1 isoform 1 protein levels were found, but a trend towards lower levels of the 170 kd Pcdh1 isoform 2 protein band (p = 0.052, Figure 3C and 3D, left panels) was observed by western blot after 5 days of smoke exposure.

In order to evaluate the reproducibility of our results, we performed an independent replication of the sub-chronic smoke exposure model. One RNA sample of the air-exposed group was excluded due to insufficient RNA quality. We detected one outlier in the smoke exposed group, both for exon 1–2 and exon 3–4 expression and tested for differences between smoke and air exposed mice with and without outliers. We observed a trend towards lower *Pcdh1* exon 1–2 expression (p = 0.16/p = 0.39; without/with outliers) and clearly replicated the decrease of *Pcdh1* exon 3–4 expression after smoke exposure (p = 0.007/p = 0.04; without/with outliers; results without outliers are shown in Figure 3B, right panel). When we combined the two experiments we observed very significantly reduced expression of *Pcdh1* exon 1–2 (p = 0.0015) and *Pcdh1* exon 3–4 (p<0.0001) mRNA. We were not able to replicate the trend towards lower isoform 2 protein expression, as we did not detect a difference in protein levels of all Pcdh1 isoforms in the replication experiment (Figure 3C and 3D, right panels).

**Figure 4:**
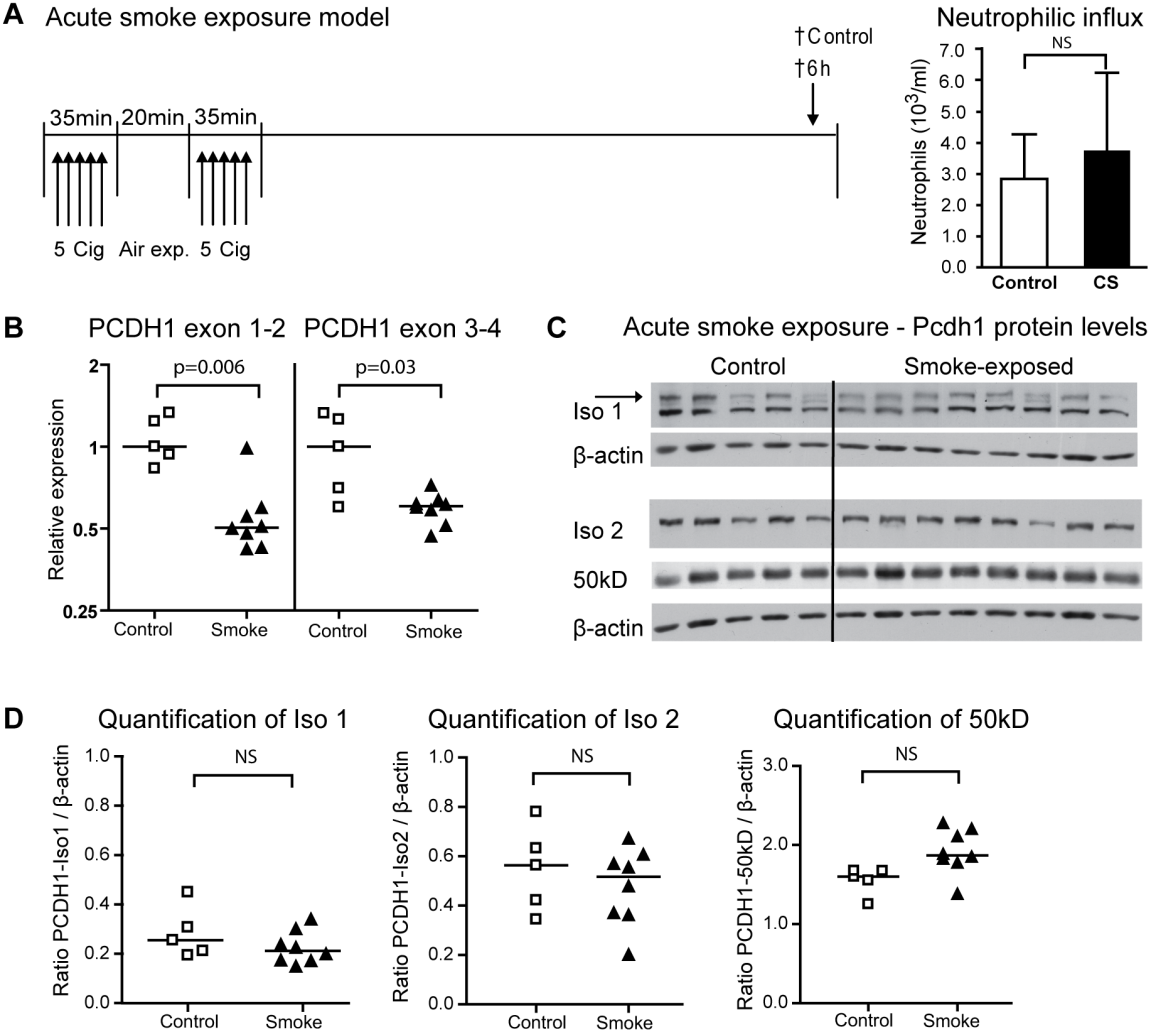
Pcdh1 mRNA expression levels decrease after acute smoke exposure. (A) In the acute smoke exposure model, mice were exposed to 10 cigarettes, with a 20 min rest period in between two sessions of 5 cigarettes, or to air as a control. Low numbers of neutrophils were observed in the lung, 6 h the last exposure. (B) Pcdh1 exon 1–2 and 3–4 mRNA expression levels were determined in the cigarette smoke exposed group (closed symbols) and compared to the air-exposed group (open symbols). (C) Western blot of Pcdh1 isoform 1 (iso 1, EP78-antibody, specific band indicated with an arrow), isoform 2 (iso 2, EP76-antibody) and the 50 kD fragment (EP76-antibody) in lung homogenate of mice exposed to cigarette smoke (smoke) or air (control). (D) Densitometric quantification of Pcdh1 expression levels of full-length isoform 1 and 2, and the 50 kD fragment, relative to β-actin. Cig = cigarette; † = time-point at which mice are sacrificed; CS = cigarette smoke.

To test whether the down regulation of *Pcdh1* mRNA expression levels was a direct effect of CS-exposure, we also analyzed *Pcdh1* expression shortly after an acute CS-exposure, in which no significant neutrophilic influx was observed (Figure 4A). Remarkably, already 6 hours after CS-exposure, a 2-fold (p = 0.006) decrease in exon 1–2 expression levels and a 1.6-fold (p = 0.03) decrease in exon 3–4 expression levels was observed (Figure 4B), suggesting that *Pcdh1* expression indeed is directly regulated by CS-exposure. This difference was not paralleled by a reduced lung Pcdh1 protein expression at this time-point (Figure 4C and 4D).

### Chronic smoke exposure down regulates Pcdh1 protein levels after 2 months

**Figure 5:**
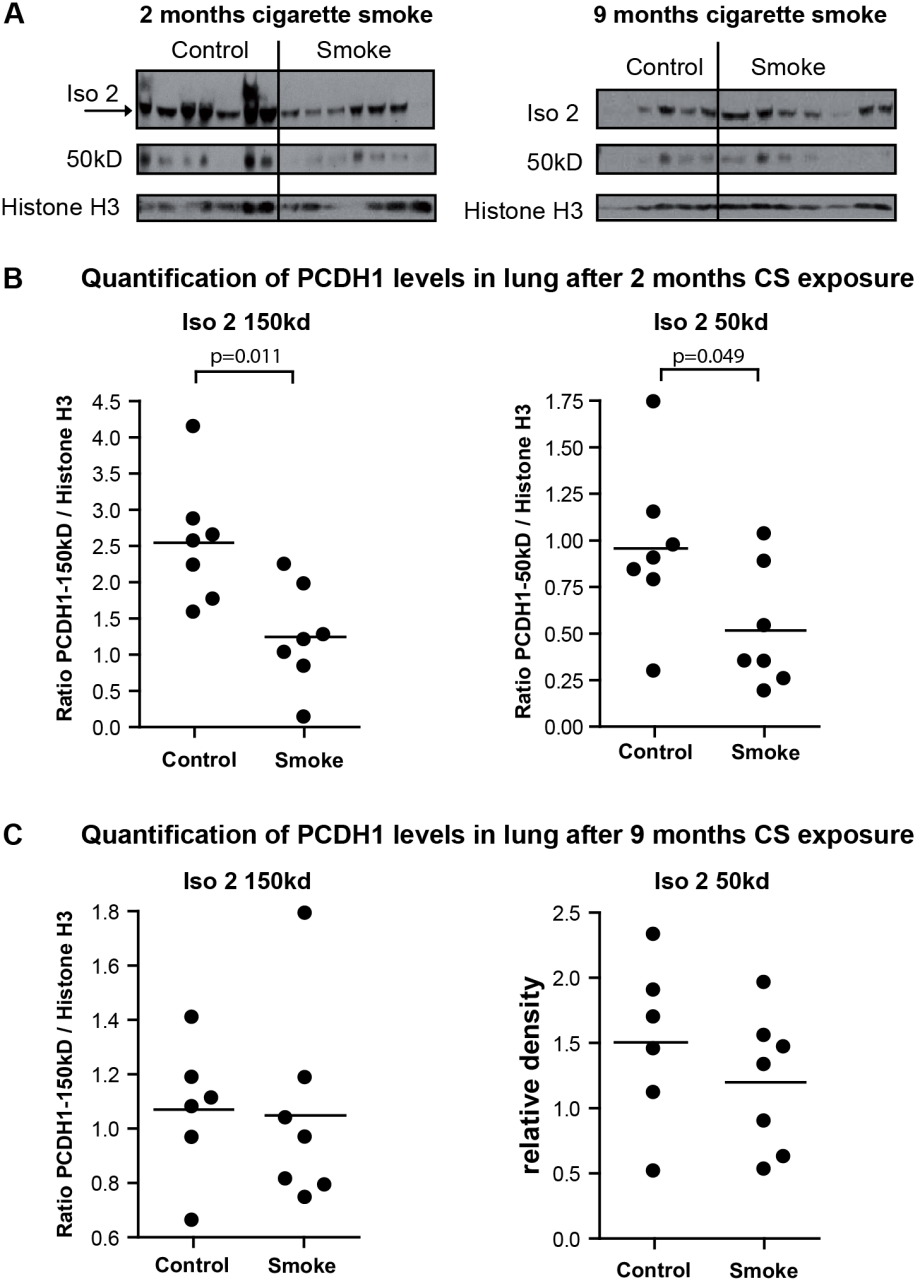
Pcdh1 protein levels are reduced after 2 months of cigarette smoke exposure. (A) Western blot of Pcdh1 isoform 2 (iso 2, EP76-antibody) and the 50 kD fragment (EP76-antibody) in lung homogenate of mice exposed to cigarette smoke (smoke) or air (control) for 2 months (left panel) or 9 months (right panel). (B) Densitometric quantification of Pcdh1 protein levels of full-length 2 and the 50 kD fragment, relative to Histone H3, after 2 months of CS exposure. (C) Densitometric quantification of Pcdh1 protein levels of full-length 2 and the 50 kD fragment, relative to Histone H3, after 9 months of CS exposure. Cig = cigarette; † = time-point at which mice are sacrificed; CS = cigarette smoke.

Next, we aimed to test whether the observed effects of CS exposure on Pcdh1 isoform 2 expression levels in lung tissue were retained after chronic smoke exposure. To this end, we analyzed lung tissue of A/J mice exposed for 5 days a week to cigarette smoke for 2 months and 9 months, compared to air-treated control mice (Figure 5). Here, we find that both the 150 kd full-length (p = 0.011) and the 50 kd band (p = 0.049) of Pcdh1 isoform 2 were significantly reduced in lung tissue after 2 months of CS exposure compared to control treatments (Figure 5A, B) while after 9 months of CS exposure, no differences in Pcdh1 protein levels in lung tissue could be detected compared to air control-treatment (Figure 5A, C).

## Discussion

In this study, we provide a full characterization of the expression pattern of Pcdh1, the murine homologue of PCDH1, a novel AHR susceptibility gene identified in families exposed to environmental cigarette smoke. We assessed Pcdh1 expression in the mouse lung and its regulation *in vivo* in both short-term and chronic CS exposure models. Here, we show that the mouse and human genes for *Protocadherin-1* are highly homologous, both at the nucleotide and at the amino-acid level, validating the mouse as a relevant model to study Pcdh1 function. In addition to the two annotated isoforms of Pcdh1 that differ in their cytoplasmic signalling domains, we observed expression of a novel Pcdh1 isoform, characterized by the lack of extracellular and transmembrane domains, whilst containing all of the intracellular domains also present in isoform 2. Exposure of mice to cigarette smoke induced a rapid and strong reduction of Pcdh1 mRNA expression in lung tissue within 6 hours. Moreover, exposure of mice to CS for 5 days induced a strongly reduced level of *Pcdh1* mRNA expression in the lung, which in one out of two experiments performed was also observed at the protein level for Pcdh1 isoform 2. The reduced protein levels of Pcdh1 isoform2 are retained in chronic CS exposure models after 2 months, but are no longer observed after 9 months of CS exposure.

When comparing the gene-structure of *Pcdh1* to human *PCDH1* [13], we observed a striking homology between the two; with 87% at the nucleotide level and 96% at the amino-acid level (Figure 1A, Figure S1). Moreover, the low expression levels of alternative 5′exons (exon m-1b), the gap in exon 4 [13], [16] resulting in deletion of conserved CM2-domains, and the novel exon 5 are also conserved between man and mouse [13]. In addition to the high level of conservation we obtained evidence for a third *Pcdh1* isoform that initiates transcription from within exon 2 and contains a CTG start-codon [24] within a conserved KOZAK sequence (Figure 1A). Overexpression of this isoform induced expression of 40-and 50 kD PCDH1 proteins that were only detected by the antibody generated against the intracellular domain encoded by exon 4 also present in isoform 2 (EP76-antibody). These results suggest that in mouse lung a third Pcdh1 protein isoform is expressed containing intracellular domains, but not extracellular cadherin repeats. Its function may involve cell signalling rather than cell adhesion, which will be subject of future investigations. We were not able to identify the exact cell-types that express this novel isoform, using the combination of approaches employed by us. The 16HBE bronchial epithelial cell-line however, was shown to display a 50 kd band on Western blot that could correspond to this novel isoform [13], but it might also represent a posttranslational modification of the full-length isoform 2 protein as has been observed for other protocadherins [25].

*PCDH1* was first identified as a susceptibility gene for AHR in families exposed to environmental tobacco smoke [12]. The human chromosomal region 5q31–33, containing *PCDH1*, was previously linked to AHR and asthma in 95 ETS exposed families. In this study, ETS exposure was defined by a significant smoking history of the proband of the asthma family. Associations of *PCDH1* gene-variants with AHR were further investigated in four populations of children exposed to ETS *in utero* or in early life. *PCDH1* gene-variant rs3822357 was associated with AHR in ETS exposed children in one population only [12]. The effect of genetic variation of the AHR susceptibility gene *PCDH1* on its expression and the putative interaction with cigarette smoke exposure as an environmental factor is currently unknown.

In the current study, we analyzed the regulation of Pcdh1 expression in experimental mouse models of cigarette smoke exposure. We observed that CS-exposure strongly reduced *Pcdh1* mRNA expression levels, as determined in whole lung homogenate. Since loss of *Pcdh1* expression is already observed as early as 6 hours after CS-exposure, *Pcdh1* expression might in fact be regulated directly by CS-exposure as this time-point precedes significant influx of inflammatory cells in the airways. Importantly, we replicated the reduction of *Pcdh1* mRNA expression levels after 4 days CS exposure, and we were able to detect down-regulation of Pcdh1 isoform 2 protein levels in lung tissue in this sub-chronic CS exposure model in one out of two experiments. Importantly, the observed down-regulation of Pcdh1 isoform 2 protein levels was retained after 2 months of chronic CS exposure.

Remarkably, in some cases we detect altered expression levels of Pcdh1 mRNA in absence of altered Pcdh1 protein levels. This apparent lack of consistent results between mRNA and protein data for the effect of CS exposure on Pcdh1 expression levels in lung tissue might be the result of differences in sensitivity between the qRT-PCR assay used to detect mRNA levels and the western blot assay used to detect protein levels. Alternatively, posttranslational modifications of the Pcdh1 protein might affect protein stability, resulting in a half-life that significantly exceeds that of the mRNA, indicating that changes in mRNA levels might only translate into altered protein levels at a relatively late time-point. All-in-all, the fact that CS exposure reproducibly induces loss of Pcdh1 expression in all three short-term exposure models and in the 2 months chronic CS exposure model presented here does indicate that CS exposure is a relevant environmental factor acting on Pcdh1 expression levels. These data are very relevant to the interpretation of the original identification of PCDH1 as an AHR gene in families exposed to environmental cigarette smoke [12]. Given our results, it is of interest to test whether in human lung tissue, too, PCDH1 expression levels are affected by CS exposure, and whether this is a function of the *PCDH1* polymorphisms identified in the original study. Moreover, studies addressing a putative role for PCDH1 gene polymorphisms in AHR in COPD patients might also be of considerable interest.

Remarkably, the differences in Pcdh1 isoform 2 protein levels in lung tissue between smoke- and air-exposed mice were no longer found after 9 months of CS exposure. Comparing the relative levels of Pcdh1 isoform 2 protein between the two time-points of the chronic CS exposure models indicates that this might be due to a loss of Pcdh1 protein expression over time in the air-control treated lungs, rather than a restoration of Pcdh1 protein levels in lungs of CS exposed mice (Figure 5B, C). Further research will need to disentangle whether progressive loss of Pcdh1 in occurring in aging lungs or whether any CS-induced regulation of Pcdh1 protein levels is lost at this late time-point.

Mice sub chronically exposed to cigarette smoke showed a trend towards hyperresponsiveness to methacholine in our experiments, an effect that was also observed in other studies in smoke exposed mice [26] and guinea pigs [27]. While PCDH1 was identified as a susceptibility gene for AHR originally, our data are merely associative: reduced Pcdh1 expression levels are associated with a trend towards AHR to methacholine, but no role for Pcdh1 in the regulation of AHR can be inferred from these data. Nevertheless, it will be of interest to test in future mechanistic experiments whether Pcdh1 protein has a direct protein-protein interaction with other epithelial proteins involved in the regulation of AHR, or whether loss of Pcdh1 induces a transcriptional response that induces an increased responsiveness to methacholine inhalation, for instance through decreased epithelial barrier function. CS is known to impair the epithelial barrier function, and thereby induces permeability of airway epithelium, as evidenced by a decrease in electrical [28] or trans-epithelial [3] resistance. Epithelial cells are interconnected by tight-junctions (TJ) and adherens junctions (AJ). CS exposure has been observed to delocalize the TJ adaptor molecule ZO-1 [29], and down-regulates several AJ adhesion molecules, including E-cadherin [30]. The resulting loss of TJ- and AJ stability may subsequently lead to loss of barrier function of the epithelium. As *PCDH1* was previously reported to have a role in cell-cell adhesion [31], we hypothesize that CS-induced decrease in Pcdh1 levels might contribute to the reduced epithelial barrier function after CS exposure. Since we only provide data on the association of reduced Pcdh1 expression levels after CS exposure, future mechanistic studies in for instance Pcdh1 knock-out mice will need to address whether Pcdh1 has any functional role in the CS-induced response by the airway epithelium, including loss of epithelial barrier function.

In conclusion, our data show that Pcdh1 is strongly conserved between mouse and man. Furthermore, our data are the first to show that *Pcdh1* mRNA expression is strongly regulated by CS-exposure, both in acute and chronic exposure models. Future studies on the function of Protocadherin-1, using novel knockout and/or transgenic approaches, and its interaction with environmental factors such as CS exposure are required to provide novel insights into the origins of airway hyperresponsiveness.

## Supporting Information

Figure S1 **Human PCDH1 and mouse Pcdh1 protein homology.**
(PDF)
