# Supplementary material for: Mouse Protocadherin-1 Gene Expression Is Regulated by Cigarette Smoke Exposure In Vivo
Source: PLoS One. 2014 Jul 3;9(7):e98197. doi: 10.1371/journal.pone.0098197 (PMC4081120; doi:10.1371/journal.pone.0098197)
Supplement: Figure S1 — Human PCDH1 and mouse Pcdh1 protein homology. (PDF) [file pone.0098197.s001.pdf]

Supplementary Figure E1

Human PCDH1 and mouse Pcdh1 protein homology:

```
SeqA Name         Len(aa)  SeqB Name          Len(aa)  Score
=============================================================
1 Human-Isoform 1  1060     2   Mouse-Isoform1   1038     96
=============================================================
CLUSTAL 2.0.12 multiple sequence alignment


Human-Isoform1  MDSGAGGRRCPEAALLILGPPRMEHLRHSPGPGGQRLLLPSMLLALLLLLAPSPGHATRV 60
Mouse-Isoform1  -----------------MGP-----LRPSPGPGGQRLLLPPLLLALLLLLAPSASHTTQV 38
                                 :**     ** ************.:***********..*:*:*

Human-Isoform1  VYKVPEEQPPNTLIGSLAADYGFPDVGHLYKLEVGAPYLRVDGKTGDIFTTETSIDREGL 120
Mouse-Isoform1  VYKVPEEQPPNTLIGSLAADYGLPDVGHLYKLEVGAPYLRVDGKTGDIFTTETSIDREGL 98
                ******************************:*************************************

Human-Isoform1  RECQNQLPGDPCILEFEVSITDLVQNGSPRLLEGQIEVQDINDNTPNFASPVITLAIPEN 180
Mouse-Isoform1  RECQNQIPGNPCILEFEVSITDLVQNGSPRLLEGQIEVQDINDNTPNFASPVITLAIPEN 158
                ******:**:**************************************************

Human-Isoform1  TNIGSLFPIPLASDRDAGPNGVASYELQAGPEAQELFGLQVAEDQEEKQPQLIVMGNLDR 240
Mouse-Isoform1  TNIGSLFPIPLATDRDAGPNGVASYELQAGPEAQELFGLQVAEDQEEKQPQLIVMGNLDR 218
                ************:***********************************************

Human-Isoform1  ERWDSYDLTIKVQDGGSPPRASSALLRVTVLDTNDNAPKFERPSYEAELSENSPIGHSVI 300
Mouse-Isoform1  ERWDSYDLTIKVQDGGNPPRASSALLRVTVLDTNDNAPKFERPTYEAELSENSPIGHSVI 278
                ****************.**************************:****************

Human-Isoform1  QVKANDSDQGANAEIEYTFHQAPEVVRRLLRLDRNTGLITVQGPVDREDLSTLRFSVLAK 360
Mouse-Isoform1  QVKANDSDQGANAEIDYTFHQAPEVVRRLLRLDRNTGLITVQGPVDREDLSTLRFSVLAK 338
                ***************:********************************************

Human-Isoform1  DRGTNPKSARAQVVVTVKDMNDNAPTIEIRGIGLVTHQDGMANISEDVAEETAVALVQVS 420
Mouse-Isoform1  DRGATPKSARAQVVVTVKDMNDNAPTIEIRGIGLVTHQDGMANISEDVAEETAVALVQVS 398
                ***:.*******************************************************

Human-Isoform1  DRDEGENAAVTCVVAGDVPFQLRQASETGSDSKKKYFLQTTTPLDYEKVKDYTIEIVAVD 480
Mouse-Isoform1  DRDEGENAAVTCVVAGDVPFQLRQASETGSDSKKKYFLQTTTPLDYEKVKDYTIEIVAVD 458
                ************************************************************

Human-Isoform1  SGNPPLSSTNSLKVQVVDVNDNAPVFTQSVTEVAFPENNKPGEVIAEITASDADSGSNAE 540
Mouse-Isoform1  SGNPPLSSTNSLKVQVVDVNDNAPVFTQSITEVAFPENNKPGEVVAEVTASDADSGSNAE 518
                *****************************:**************:**:************

Human-Isoform1  LVYSLEPEPAAKGLFTISPETGEIQVKTSLDREQRESYELKVVAADRGSPSLQGTATVLV 600
Mouse-Isoform1  LVYSLEPEPAAQGLFTISPENGEIRVKTSLDREQRDSYELKVVAADRGSPSLQGTATVLV 578
                ***********:********.***:**********:************************

Human-Isoform1  NVLDCNDNDPKFMLSGYNFSVMENMPALSPVGMVTVIDGDKGENAQVQLSVEQDNGDFVI 660
Mouse-Isoform1  NVLDCNDNDPKFMLSGYNFSVMENMPALSPVGMVTVIDGDKGENARVQLSVEQDNGDFVI 638
                *********************************************:**************

Human-Isoform1  QNGTGTILSSLSFDREQQSTYTFQLKAVDGGVPPRSAYVGVTINVLDENDNAPYITAPSN 720
Mouse-Isoform1  QNGTGTILSSLSFDREQQSTYTFQLKAVDGGVPPRSAYVGVTINVLDENDNAPFITAPSN 698
                *****************************************************:******

Human-Isoform1  TSHKLLTPQTRLGETVSQVAAEDFDSGVNAELIYSIAGGNPYGLFQIGSHSGAITLEKEI 780
Mouse-Isoform1  TSHRLLTPQTRLGETVSQVTAEDIDSGINAELTYSIAGGNPYGLFQIGSHSGAITLEKEI 758
                ***:***************:***:***:**** ***************************

Human-Isoform1  ERRHHGLHRLVVKVSDRGKPPRYGTALVHLYVNETLANRTLLETLLGHSLDTPLDIDIAG 840
Mouse-Isoform1  ERRHHGLHRLVVKVSDRGKPPRYGTALVHLYVNETLANRTLLETLLVHSLDTPLDIDIAG 818
                ********************************************** *************

Human-Isoform1  DPEYERSKQRGNILFGVVAGVVAVALLIALAVLVRYCRQREAKSGYQAGKKETKDLYAPK 900
Mouse-Isoform1  DPEYERSKQRGNILFGVVAGVVAVALLIALAVLVRYCRQREAKSGYQAGKKETKDLYAPK 878
                ************************************************************

Human-Isoform1  PSGKASKGNKSKGKKSKSPKPVKPVEDEDEAGLQKSLKFNLMSDAPGDSPRIHLPLNYPP 960
Mouse-Isoform1  PSGKAAKGSKSKGKKSKSPKPVKPVEDEDDTGLQKSLKFNLMSDAPGDSPRIHLPLNYPP 938
                *****:**.********************::*****************************

Human-Isoform1  GSPDLGRHYRSNSPLPSIQLQPQSPSASKKHQVVQDLPPANTFVGTGDTTSTGSEQYSDY 1020
Mouse-Isoform1  GSPDLGRHYRSNSPLPSIQLQPQSPSASKKHQVVQDLPPANTFVGTGDTTSTGSEQYSDY 998
                ************************************************************

Human-Isoform1  SYRTNPPKYPSKQVGQPFQLSTPQPLPHPYHGAIWTEVWE 1060
Mouse-Isoform1  SYRTNPPKYPSKQVGQPFRLSTPQPPPHPYHGAIWTEVWE 1038
                ******************:****** **************
```

EP78 peptide

```
SeqA Name           Len(aa)  SeqB Name         Len(aa)  Score
=============================================================
1  Human-Exon3-4   204       2    Mouse-Exon3-4  205      96
=============================================================
CLUSTAL 2.0.12 multiple sequence alignment


Human-Exon3-4    LPHRRVTFSATSQAQELQDPSQHSYYDSGLEESETPSSKSSSGPRLGPLALPEDHYERTT 60
Mouse-Exon3-4    LPHRRVTFSATSQAQELQDPSQHSYYDSGLEESETPSSKSSSGPRLGPLALPEDHYERTT 60
                 ************************************************************

Human-Exon3-4    PDGSIGEMEHPENDLRPLPDVAMTGTCTRECSEFGHSDTCWMPGQSSPSRRTKSSALKLS 120
Mouse-Exon3-4    PDGSIGEMEHPENDLRPLPDVAMTGTCTRECSEFGHSDTCWMPGQSSPSRRTKSSALKLS 120
                 ************************************************************

Human-Exon3-4    TFVPYQDRGGQEPAGAGSPSPPEDRNTKTAPVRLLPSYSAFSHSSHDSCKDSATLEEIPL 180
Mouse-Exon3-4    TFVPYQDRGGQEPAGAGSPSPPEDRNTKTAPVRLLPSYSAFSHSSHDSCKDSATLEEIPL 180
                 ************************************************************

Human-Exon3-4    TQTSDFPPAATPASAQT-AKREIYL 204
Mouse-Exon3-4    TQTSDFPPTATPASAQTAAKREIYL 205
                 ********:******** *******
```

EP76 peptide
